# Relationship between family caregiver quality of life and the care provided to people living with dementia: protocol for a mixed methods study

**DOI:** 10.3934/publichealth.2020025

**Published:** 2020-05-11

**Authors:** Afeez Abiola Hazzan, Patti Follansbee, Jason Dauenhauer, Alexandra E Completo

**Affiliations:** 1 Department of Healthcare Studies, the College at Brockport, State University of New York, 350 New Campus Drive, Brockport, New York 14420, USA; 2 Department of Social Work, the College at Brockport, State University of New York, 350 New Campus Drive, Brockport, New York 14420, USA

**Keywords:** family caregiver, quality of life, quality of care, level of care, dementia, Alzheimer's disease, aging, caregiving

## Abstract

**Objective:**

Family caregivers of people with dementia perform duties that are important for maintaining their loved one's overall well-being. However, it is not yet clear how these caregivers' quality-of-life affects their ability to meet the care demands of their loved ones. The purpose of this study is to utilize a mixed methods approach in investigating how family caregiver quality-of-life affects the care provided to people with dementia. Family caregivers will be recruited from the Rochester, New York area to participate in focus groups or one-on-one interviews. In addition to the qualitative data obtained, caregivers will complete questionnaires regarding their own quality-of-life (e.g., health status, socioeconomic) as well as the care provided to their loved ones with dementia (e.g., how the care provided meets the needs of the care recipient, time spent). A convergent mixed methods approach will be used to analyze the qualitative and quantitative data obtained.

**Results:**

Data from the interviews will be transcribed verbatim and then analyzed qualitatively. Quantitative data from the questionnaires will be analyzed using IBM SPSS Statistics software. A convergent mixed methods approach will be applied to the datasets to help shed light on the relationship between family caregiver quality-of-life and the care provided to people living with dementia. Understanding of this relationship will make it possible to develop initiatives that better address caregiver needs.

## 1 Introduction

Primary informal caregivers are mainly responsible for the care of people with dementia including Alzheimer's disease (AD). Caregivers perform a variety of duties ranging from shopping for their loved ones' groceries, helping with medications, managing finances and legal affairs, guarding against wandering, and helping with basic and instrumental activities of daily living [1]. However, it is important to note that the caregiving role becomes more demanding as the disease progresses over time, and studies have shown that the quality-of-life (QoL) experienced by dementia caregivers is lower than the QoL of caregivers for persons who do not have this disease [2].

Although several studies have provided evidence for positive aspects of caregiving including lower depression and higher life satisfaction rate, the majority of caregivers find this role to be stressful [2]–[4]. In addition to the stressful nature of caregiving, the care recipient's condition tends to decline over time, making the caregiving role more demanding [5]. This may in turn affect the caregiver's ability to meet their loved ones' care needs, resulting in a decline in the quality of care provided to the care recipient as the disease progresses.

However, there has been no research conducted to directly investigate whether lower caregiver QoL (both pre-existing QoL or changing QoL as a function of caregiving) affects the level or quality of care that caregivers provide to persons with dementia. In a randomized controlled trial assessing the effectiveness of the STrAtegies for RelaTives (START) intervention in the short and long term, the researchers found that an eight-session manual-based coping intervention resulted in decreased anxiety and depression levels for caregivers [6]. Although the researchers also found evidence for improved QoL among caregivers, this did not result in improvement of the QoL of the person living with dementia.

In a published systematic review examining the relationship between caregiver QoL and the quality of care provided [7], we found only one study that was somewhat relevant [8]. However, this single study did not examine the association between QoL and level or quality of care provided to people with dementia. It suggested that the main reason for an absence of research into this topic was the absence of an instrument (i.e. questionnaire) for measuring quality of care provided to care recipients with dementia [7].

## 2 Materials and methods

The study will utilize a convergent mixed methods approach to answer the following research question:

What is the relationship between caregiver quality-of-life and the care provided to persons with dementia?

A convergent mixed methods design involves the collection, analysis, and comparison of both qualitative and quantitative data as part of the same study [9]. To be consistent with this type of mixed methods design, both quantitative and qualitative data will be collected at the same time as part of the same interview session. However, the quantitative and qualitative data will be analyzed separately and the results used to confirm or disconfirm each other [9],[10]. The convergent mixed methods approach also makes it possible to maximize different types of information (for example views of focus group participants and scores from objective questionnaires) to generate results that are richer than what the individual method would have allowed [9],[10].

Further, the convergent mixed methods design permits the efficient collection of data from the same number of participants on both the qualitative and quantitative components since the intent is to make a comparison between the two types of data [9],[10]. Purposeful sampling will be used to select approximately 25 family caregivers from the Rochester, New York area to participate in focus groups or one-on-one interviews (depending on caregiver availability). The sample size of approximately 25 family caregivers selected through this sampling approach will make it possible to collect a sufficient database of qualitative and quantitative information to permit an in-depth understanding of the relationship between family caregiver quality of life and the care provided to people living with dementia [10]. The same participants will be used for both the qualitative and quantitative components of the study. Although caregivers are usually close relatives (e.g., spouses, adult children) of the person with dementia, anyone who provides uncompensated care for at least four hours per week to a person living with dementia will be eligible for inclusion in this study [2].

Caregiver serving organizations (e.g. Lifespan of Greater Rochester) in the Rochester, New York area will be approached to help identify family caregivers meeting the inclusion criteria. These organizations will send recruitment documents (recruitment letter, e-mail) to potential family caregivers inviting them to participate in the study, and interested participants will be asked to contact the principal investigator (AH). Interviews will take place on-site at the caregiver serving organization's premises and on SUNY Brockport's campus. It should be noted that due to current social distancing restrictions related to the COVID-19 pandemic, focus groups and interviews may be conducted through online video or voice/telephone conferencing. Factors influencing this decision will be made individually with each caregiver participant depending on their access and comfortability with technology. Recruitment materials will include information about preferred options of interaction available to participants. The principal investigator (AH) will facilitate the interviews, and each focus group will have six to eight interviewees [9]. The principal investigator (AH) has extensive experience with focus group interviewing, and has published peer-reviewed studies utilizing qualitative methods [5],[11]. He will note any personal biases or preferences, and will not allow these to affect the research. Each participant will be given a $20 gift card at the end of their interview as compensation for their time and travel expenses. Informed consent will be obtained from participants at the beginning of each interview (focus group or one-on-one). During the focus group interviews, participants will answer questions such as: *Do you receive assistance from other people in providing care for your loved one? Are you always able to meet your loved ones' care needs? etc.*

In addition to the qualitative data obtained during these research interviews, caregivers will complete questionnaires regarding their own QoL (for example, health status, employment, etc.) as well as the care (e.g. how the care provided meets the needs of the care recipient, time spent) provided to their loved ones. Family caregiver will complete SF-36, a validated 36-item questionnaire that captures the following eight areas of QoL: vitality, physical functioning, bodily pain, general health perceptions, physical role functioning, emotional role functioning, social role functioning, and mental health [12]. Data from the focus groups and/or interviews will be transcribed verbatim and then analyzed qualitatively by coding the information into major ideas or themes from the perspective of the participants [9]. Quantitative data from the questionnaires will be analyzed using IBM SPSS Statistics version 25 (IBM Corporation, Armonk, NY). The level of statistical significance for the quantitative analysis will be set at p < 0.05. The mixed methods data analysis will involve integrating the qualitative and quantitative results by doing a side-by-side comparison of the two forms of data within the discussion section [9].

## 3 Results and discussion

In order to reveal the demographic characteristics of caregivers used in this study (e.g., average age, income, relationship types), descriptive statistics will be computed using data obtained from the interviews and questionnaires. Results from the qualitative and quantitative analyses will identify the components of quality of life that may be important in influencing the care provided by family caregivers. Overall, the study results will help shed light on the relationship between family caregiver QoL and the quality of care provided to people with dementia.

Considering the rapid aging of the U.S. population [1],[2], this study will address an important topic. Once we understand how caregiver QoL affects the care provided to persons with dementia, it will be possible to develop initiatives that better address caregiver needs. These initiatives may include an expansion of respite care for persons with dementia or the provision of specialized training to help caregivers better cope with the impact of the disease on their own QoL. This could ultimately minimize long-term disability and lessen the impact of dementia on the society at large.
